# Unveiling the Immediate Impact of Prechtl’s General Movement Assessment Training on Inter-Rater Reliability and Cerebral Palsy Prediction

**DOI:** 10.3390/neurosci5030019

**Published:** 2024-07-23

**Authors:** Namarta Kapil, Bittu Majmudar-Sheth, Alexa Celeste Escapita, Tara Johnson

**Affiliations:** 1 Department of Neurobiology and Developmental Sciences, University of Arkansas for Medical Sciences, Little Rock, AR 72205, USA; kapiln@wustl.edu (N.K.); acescapita@uams.edu (A.C.E.); 2 Department of Pediatrics, Division of Pediatric Neurology, University of Arkansas for Medical Sciences, Little Rock, AR 72205, USA; bmajmudar@iuhealth.org; 3 Department of Neurology, Indiana University School of Medicine, Riley Hospital for Children at IU Health, Indianapolis, IN 46202, USA

**Keywords:** cerebral palsy, Prechtl’s general movement assessment (GMA), high-risk infants, inter-rater reliability

## Abstract

Prechtl’s General Movement Assessment (GMA) is a qualitative video assessment that is an early predictor of cerebral palsy (CP) in infants. GMA raters undergo two levels of training: basic and advanced. Using a retrospective study design, we evaluated the impact of the GMA training level on three key measures: inter-rater reliability (IRR), predictability for a later CP diagnosis, and generalizability for both medically trained and non-medically trained raters. As part of a larger study on former level-IV neonatal intensive care unit (NICU) infants, participants had a GMA video at 3–4 months of corrected age with parental consent. Four GMA raters with basic training interpreted the videos. Subsequently, two raters underwent advanced training and reinterpreted the videos. We evaluated IRR with Gwet’s AC1 coefficient, CP prediction with logistic regression, and generalizability with Gwet’s Linearization method and McNemar’s test. Following basic GMA training, all four raters had moderate to almost perfect agreement. The CP predictability of the raters’ individual GMA scores ranged from 73% to 81%. Advanced GMA training significantly improved IRR and enhanced CP predictability. GMA rater proficiency was similar regardless of medical training. Advanced GMA training improved readers’ capabilities to correctly identify abnormal movement patterns and aid in early CP diagnosis.

## 1. Introduction

Cerebral palsy (CP) is a disorder of movement and posture that leads to activity limitations [1]. It arises from non-progressive brain injuries during early brain development and affects nearly four per thousand children in the United States [1,2]. Despite the Centers for Disease Control and Prevention’s recognition of CP as one of the primary motor disorders affecting children, the average age of diagnosis for CP still remains approximately two years of age [3,4,5]. One of the tools that has gained considerable recognition to aid in early CP diagnosis is Prechtl’s General Movement Assessment (GMA) [3,6,7].

Developed in Europe, the GMA is a qualitative video assessment that is performed by trained raters who classify infants’ movement patterns. The assessment is based on visual Gestalt perception of age-specific normal and abnormal general movement patterns [6,8,9]. General movements (GMs) are spontaneous movements characterized by a diverse and intricate range of movements that have a specific spatial–temporal organization [6,7]. GMs can be detected from early fetal life until the intentional and anti-gravity movements become predominant at 3–5 months post-term age. GMs serve as a means to evaluate the integrity of the immature nervous system. Such movements include fidgety movements, which are most apparent from 3–5 months post-term age. During the fidgety period, the infant has moderate speed, variable acceleration, and small-amplitude movements involving the entire body. Infants with excessive fidgety movements (abnormal movements) or absence of fidgety movements (absent fidgety movements) are highly likely to have atypical motor development [6,7]. Abnormal fidgety movements are very rare and possess a low predictive value for a later diagnosis of CP. Infants with abnormal fidgety movements may either follow a normal developmental trajectory or have coordination and fine manipulative disabilities. Absent fidgety movements, on the other hand, have demonstrated high predictability for later neurological deficits, specifically CP [6,7,10]. Generally, variability of movements is regarded as a favorable prognostic sign.

GMA only requires a brief 2−5 min observation of an infant’s spontaneous movement, without the need for physical contact by the assessor. This makes the evaluation significantly straightforward to conduct, compared to most other assessments for neurological development [10,11].

Many standard assessment tools require the examiner to physically interact with the child by providing visual, auditory, vestibular, and tactile stimuli to the infant and then assessing the infant’s responses to stimuli. Although the examiner does not need to perform complicated maneuvers to assess the child, the GMA can only be performed by certified raters who have undergone specialized GMA training [11]. This training is divided into two levels: basic and advanced. In basic GMA training, learners are only taught how to differentiate between fidgety, absent, and abnormal fidgety movements as a whole [12]. Advanced training offers learners more in-depth instruction on accurately assessing the components of general movements, such as speed, amplitude, rotations, and postures, to correctly distinguish between normal vs. abnormal movement (absent/abnormal) patterns [13]. Following training, the ongoing use of GMA and periodic recalibrations is also recommended [11].

Despite the effectiveness of GMA, the adoption of GMA in daily clinical practice faces some challenges. Firstly, the cost for each four-day training session ranges from USD 950–975, excluding travel and hotel accommodations [14,15]. The basic course has limited availability with few slots [14]. The availability of the advanced GMA course is even more limited, occurring only once or twice per year in the United States, with more limited slot availability [15]. Consequently, physicians often attend only the basic GMA training and forgo advanced training due to resource constraints or unavailability during advanced training dates.

Additionally, while the GMA’s inter-rater reliability (IRR) has consistently demonstrated excellence in studies across various sites, it is important to note that these studies primarily involved experienced raters who had undergone advanced GMA training and possessed years of expertise [11,16,17,18,19]. In instances where more novice raters assessed the videos, inter-rater reliability values tended to be relatively lower [16,20]. However, there is a notable gap in the literature regarding studies that evaluate both inter-rater reliability and CP diagnosis prediction immediately after successful completion of basic or advanced GMA training. GMA reader skill proficiency varies among individuals and may be influenced by factors such as years of expertise [20]. There is a need for studies emphasizing the effectiveness of GMA immediately after training, as opposed to evaluations conducted after years of practical experience. Such studies can provide valuable insights for physicians who incorporate GMA into clinical practice promptly after completing their training.

Therefore, the aim of this study was to bridge this gap in the literature by (1) assessing (a) IRR post-basic GMA training and (b) the improvement in IRR post-advanced GMA training, (2) evaluating (a) prediction of CP post-basic GMA training and (b) the improvement in prediction of CP post-advanced GMA training, and (3) exploring the potential influence of a medical background on the proficiency of a GMA reader through comparisons between medically and non-medically trained GMA raters.

## 2. Materials and Methods

### 2.1. Setting and Participants

As part of a prospective cohort study on former level-IV neonatal intensive care unit (NICU) infants at Arkansas Children’s Hospital, participants had a GMA assessment at 3–4 months of corrected age with parental consent. The cohort was identified as infants who had an inpatient neurologic consultation from May 2019 to October 2020 for conditions that included the following: <37-week gestational age, seizures, positional plagiocephaly, hypoxic ischemic encephalopathy (HIE), Down syndrome, hydrocephalus, and abnormal head imaging on brain magnetic resonance imaging (MRI) or head ultrasound, with findings that include intracranial bleeding (grade 2–4 intraventricular hemorrhage (IVH), hematomas, and other hemorrhages) and white matter changes such as periventricular leukomalacia (PVL) and encephalomalacia. Their developmental outcomes at 2–3 years of age were available as part of that study.

We conducted a retrospective nested case–control study involving this cohort. We defined cases as participants who had been diagnosed with CP and designated all other subjects as controls.

### 2.2. GMA Raters

The four GMA raters (PhD1, PhD2, MD1, and MD2) successfully completed a 4-day basic course, and two of them (PhD1 and PhD2) also successfully completed a 4-day advanced training course. Raters PhD1 and PhD2, both PhD candidates, lack prior medical and clinical experience, and they do not use GMA besides watching the videos from this study. MD1 and MD2, both pediatric neurologists who incorporate GMA into their clinical practice, had recently completed their basic GMA training at the time of the assessment for this study.

MD2 was the treating neurologist for these infants in the pediatric neurology follow-up clinic. She recorded the GMA videos and she was aware of the infants’ medical history. PhD1, PhD2, and MD1 were blinded to the infants’ medical history.

### 2.3. Procedure

Prior to assessing the GMA videos, the raters each calibrated themselves by viewing the educational GMA video provided by the GM Trust [21]. This video included demonstrations of normal and abnormal fidgety movements.

The GMA video recordings took place during the subjects’ pediatric neurology follow-up clinic visits. With parental consent, MD2 positioned the infant in the supine position and recorded a 2 min video for GMA interpretation using an iPad mini. Then, PhD1, who was blinded to the subjects’ medical history, uploaded these videos from the iPad mini to a secure UAMS Box, coded them, and granted access to all the raters. 

PhD1, PhD2, MD1, and MD2 each independently viewed the videos after successfully completing basic GMA training. PhD1 and PhD2 then successfully completed advanced GMA training, and they subsequently reinterpreted the videos. PhD1 had a 2-year gap between viewings and PhD2 had a 1-year gap between viewings. Neither PhD1 nor PhD2 had utilized GMA outside of these video assessments.

### 2.4. GMA Scoring

The raters coded movements as either “fidgety” (F), “abnormal fidgety” (AF), or “absent fidgety” (F-) [17,18,22]. For analytical purposes, PhD1 dichotomized the results into two categories: present (normal or abnormal (exaggerated)) or absent (sporadic or absent). CP outcomes were unavailable or unknown to all raters at the time of video analysis.

### 2.5. Statistical Analysis

In this retrospective study, we categorized participants diagnosed with CP as cases, while all other participants were designated as controls. Some of the control participants had typical development and others had developmental delay (DD) with no neurologic signs of CP.

Demographic characteristics were reported as frequency and percentage (%) for categorical variables and mean and standard deviation for continuous variables. To assess IRR between GMA raters, Gwet’s AC1 coefficient was utilized in lieu of the widely used Cohen’s kappa. This choice was based on recent reports advocating for its use in populations like ours. Gwet’s AC1 is preferred because it is less influenced by prevalence and marginal probability than Cohen’s kappa, especially in cases involving an ordered categorical rating system [20,23,24,25].

Interpretation of the reliability coefficient followed definitions from Landis and Koch: slight agreement (0.00 to 0.20), fair agreement (0.21 to 0.40), moderate agreement (0.41 to 0.60), substantial agreement (0.61 to 0.80), and almost perfect agreement (0.81 to 1.00) [16,20].

The change in IRR for PhD1 and PhD2 after advanced GMA training was quantified using Gwet’s Linearization method [23,24,26].

Additionally, logistic regression analysis was employed to create receiver operator characteristic (ROC) curves to assess the accuracy of GMA scores from the 4 individual raters in classifying infants with and without CP. ROC plots were also utilized to illustrate the impact of advanced GMA training on the predictability of CP for both PhD1 and PhD2. To evaluate differences between non-medically trained (PhD1, PhD2) raters and medically trained raters (MD1, MD2), IRR and percent agreement were compared with Gwet’s Linearization method and McNemar’s statistical analysis, respectively.

Statistical analyses were performed using RStudio (RStudio Team, 2020, R Core Team, 2021) [27,28].

## 3. Results

A total of 98 subjects underwent GMA evaluation at 3–4 months of corrected age, with subsequent neurodevelopmental outcomes assessed at 2–3 years of age. Of the 98 subjects, 17 subjects were determined to have CP and the remaining 81 did not have CP. The demographic characteristics are depicted in Table 1.

The IRR between the raters post basic and advanced GMA training is depicted in Table 2. After basic GMA training, the IRR between raters ranged from 0.59 to 0.83, indicating a moderate to almost perfect agreement level. PhD1 and PhD2 demonstrated a statistically significant improvement (*p* = 0.046) in their IRR after advanced GMA training. Their Gwet’s AC1 coefficient improved from 0.59 after basic training to 0.76 after advanced training, indicating a shift from moderate to substantial agreement.

**Figure 1:**
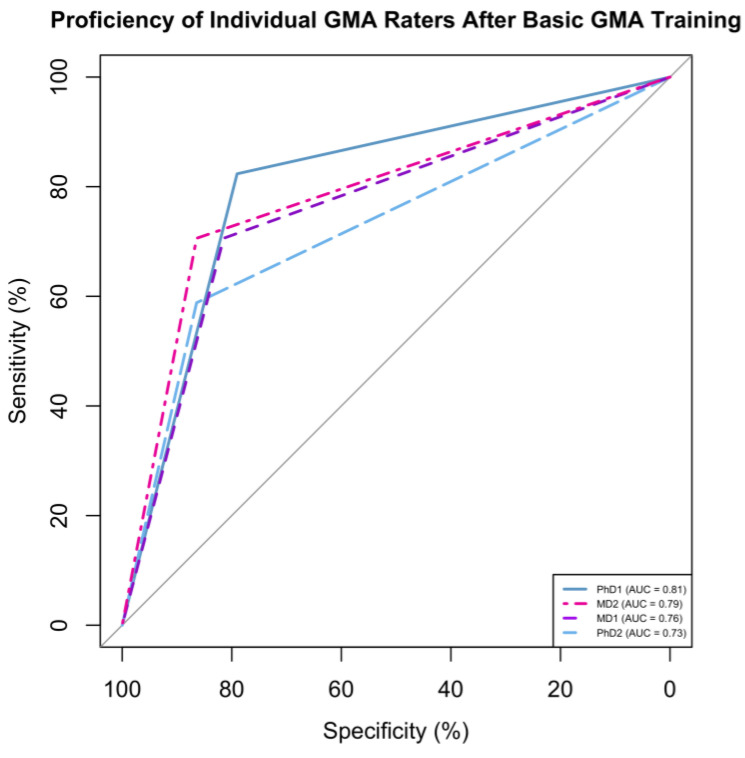
ROC plots illustrating the predictability of GMA scores for CP outcomes after basic training. These results are for each of the four raters (PhDs in blue, MDs in pink). Abbreviations: AUC: area under the curve.

**Figure 2:**
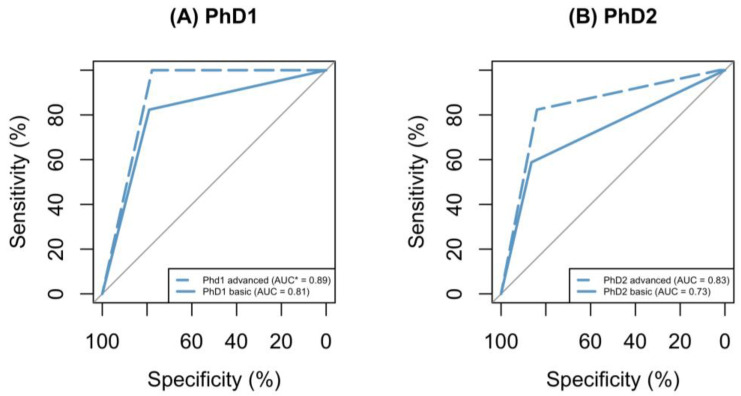
ROC plots comparing the GMA’s ability to predict CP outcomes. Pre- and post-advanced GMA training are shown by the solid line and dashed line, respectively. * Abbreviations: AUC: area under the curve.

ROC plots were generated to illustrate the predictability of GMA scores post-basic training for individual raters (Figure 1) in relation to later CP diagnosis. The area under the curve (AUC) for individual raters ranged from 73 to 81%. After completing their advanced GMA training, PhD1 and PhD2 had improvements in their AUC scores, increasing from 81% to 89% and from 73% to 83%, respectively (Figure 2).

Notably, no significant statistical differences were found between the IRR (*p* = 0.2) and the percent agreement (*p* = 0.3) between medically and non-medically trained raters.

## 4. Discussion

In this study, our objective was to unveil the impact of the GMA training level on three key aspects: IRR, predictability for a later CP diagnosis, and generalizability. We found that, after basic GMA training, there was moderate to almost perfect agreement between raters, and the predictability for CP ranged from 73% to 81%. Following advanced GMA training, there was a statistically significant improvement in the IRR for PhD1 and PhD2. Moreover, both raters exhibited enhanced predictability for CP, underscoring the positive impact of advanced GMA training. It is noteworthy that PhD1 and PhD2 engaged in GMA assessments solely for the study videos. Thus, the observed enhancements in their IRR and predictability result directly from the impact of advanced training, rather than ongoing GMA utilization in clinical practice.

Another notable observation was that MD2, despite knowing the medical history of the participants, achieved comparable results for both IRR and CP predictability compared to other raters who were blinded to medical history. Additionally, there was no statistically significant difference observed in IRR and percent agreement between medically and non-medically trained raters (PHD1, PHD2 vs. MD1, MD2). These observations of comparable results in IRR and predictability, irrespective of medical background or knowledge of clinical history, affirm the robustness and reliability of GMA in various clinical applications.

Following its introduction in 1990, GMA training courses have become increasingly prevalent in Europe. With the GMA’s growing popularity, the training courses have broadened their reach to the Americas, Australia, Asia, and South Africa [29].

After its initial introduction, GMA training was taught in a 3.5–5-day-long course. To assess the effectiveness of the GMA training, Valentin et al., conducted an evaluation of the initial 18 training courses that were held from 1997 to 2002. This study revealed that 83% of over 8000 GM assessments were correctly performed after just four to five days of training [22]. This study was published in 2005, and subsequently there have been changes in GMA training, notably the introduction of two sequential courses instead of a single course [12,13]. Therefore, more studies are needed to demonstrate the immediate effectiveness of both basic and advanced GMA training, as addressed in our study.

Our study offers new information that contributes to the existing literature on GMA. Firstly, we provide insight into the efficiency of GMA for two of its key aspects: IRR and CP prognostication immediately after both basic and advanced GMA training. Previous studies have reported almost perfect IRR (kappa > 0.8) and intra-rater agreement (kappa: 0.85–1). However, these studies were performed by members of the GM Trust who have years of experience utilizing GMA in both clinical and research settings [17,18,19,29,30]. Novice raters, on the other hand, often struggle, especially when they are interpreting difficult cases [29]. Bernhardt et al., reported that interrater agreement was only fair to substantial (kappa < 0.60) if a scorer was relatively inexperienced, regardless of the successful completion of GMA training [16]. Peyton et al. reported similar findings, utilizing Gwet’s AC1 coefficient in addition to Cohen’s kappa for assessing IRR [20]. Both of these studies had utilized experienced physiotherapists as raters, and therefore all the raters had some experience using GMA in a clinic (anywhere from less than a year to a few years) [16,20]. Our study, on the other hand, utilized novice raters who had recently completed their training prior to interpreting the GMA videos. Additionally, two of our raters were PhD students with no medical training, providing a more realistic perspective on the third key aspect of GMA, i.e., the generalizability of GMA’s reliability.

Furthermore, our study, to the best of our knowledge, is the first to evaluate the impact of advanced GMA training on enhancing GMA reading capabilities, without the confounding factor of years of expertise influencing the outcomes. These results highlight the importance of physicians and therapists, undergoing advanced GMA training for improved predictability of CP. Given that physicians and therapists rely on observations to recommend follow-up care, increased accuracy in their interpretations, as observed in our study after additional training, highlights the tangible benefits of such advanced training.

While our study presents valuable insights, it is important to acknowledge its limitations. Firstly, the retrospective study design and the single-clinic subject population may impact the generalizability of our findings. Secondly, the small sample size of GMA readers affects the robustness of our results. More studies with larger sample sizes are needed to corroborate these findings. Lastly, the physicians involved in the study were unable to undergo advanced GMA training at the time of the study. Therefore, data were analyzed using dichotomized results instead of utilizing the more nuanced “fidgety, intermittent, sporadic, absent, and abnormal” classifications [20]. This simplification was necessary due to the absence of advanced GMA training for all raters. The detailed classification is only part of the advanced training. In basic GMA training, learners are only taught how to differentiate between typical and atypical fidgety movements as a whole.

The primary reasons for the lack of advanced GMA training for the two physicians initially stemmed from the course’s unavailability due to COVID-related travel restrictions. Even after these restrictions were lifted, the increased demand for the course post-pandemic limited the chances for physicians to enroll, given the limited slot availability. Scheduling conflicts during subsequent advanced GMA course dates added another layer of impediment, preventing them from accessing this training. To address these challenges, we recommend that the GM Trust considers providing more time slots for advanced GMA training, moving beyond the current practice of offering it just once or twice per year with limited slot availability in the United States.

In conclusion, despite the small sample size of GMA raters in our study, our findings provide valuable insights into the immediate effectiveness of GMA post-training in a realistic clinical setting, where only a few physicians and therapists are trained in GMA. Notably, our study unveils the positive impact of advanced GMA training on enhancing inter-rater reliability and predictability of CP for novice GMA raters. This information holds practical significance for physicians and therapists using GMA in their clinical practice to guide their recommendations for follow-up care in high-risk infants.

## Figures and Tables

**Table 1:** Demographic characteristics.

Subject Demographics	Totals *n* = 98 (%)
Gender	
Female, *n* (%)	33 (34)
Race	
White, *n* (%)	52 (53)
Black or African American, *n* (%)	31 (32)
Other, *n* (%)	12 (12)
Unknown, *n* (%)	3 (3)
Insurance	
Medicaid, *n* (%)	73 (75)
Private, *n* (%)	23 (24)
None, *n* (%)	2 (2)
Medical History (known risk factors for CP)	
Birthweight (Kg), mean (SD)	2.63 (1.1)
Premature (<37 weeks GA), *n* (%)	49 (50)
Gestational Age, mean (SD)	35.1 (4.9)
Abnormal Brain MRI, *n* (%)	68 (69)
Abnormal Head Ultrasound, *n* (%)	50 (51)
Seizures, *n* (%)	13 (13)
Intracranial Bleeding	
Intraventricular Hemorrhage (IVH), *n* (%)	27 (28)
Hematomas (Intraparenchymal and Subdural), *n* (%)	2 (2)
Hemorrhages (Intracranial and Cerebellar), *n* (%)	5 (5)
Periventricular Leukomalacia (PVL), *n* (%)	12 (12)
Hypoxic Ischemic Encephalopathy (HIE), *n* (%)	44 (50)
Hypoxic Brain Injury, *n* (%)	1 (1)
Anoxic Brain Injury, *n* (%)	1 (1)
Positional Plagiocephaly, *n* (%)	25 (26)
Down Syndrome, *n* (%)	2 (2)
Hydrocephalus, *n* (%)	12 (12)
Encephalomalacia, *n* (%)	2 (2)

**Table 2:** Inter-rater reliability (IRR) between different combinations of raters post-basic and post-advanced GMA training.

Inter-Rater Combinations	Percent Agreement	AC1 Coefficient (95% Confidence Interval)	Interpretation
Post-Basic GMA * Training			
{PhD1, PhD2}	76%	0.59 (0.43–0.76)	Moderate
{PhD1, MD1}	90%	0.83 (0.72–0.94)	Almost perfect
{PhD1, MD2}	88%	0.80 (0.68–0.91)	Substantial
{PhD2, MD1}	76%	0.61 (0.45–0.77)	Substantial
{PhD2, MD2}	78%	0.66 (0.51–0.81)	Substantial
{MD1, MD2}	82%	0.70 (0.56–0.85)	Substantial
Post-Advanced GMA Training			
{PhD1, PhD2}	82%	0.76 (0.66–0.87)	Substantial

* Abbreviations: GMA: Prechtl’s General Movement Assessment.

## Data Availability

The data from this study will not be made available to protect the research participant’s privacy.
